# Association of child abuse and neglect training with filing reports of concern to child welfare services: a cross-sectional study

**DOI:** 10.1186/s12903-024-04222-9

**Published:** 2024-04-06

**Authors:** Nancy Birungi, Karin Goplerud Berge, Anne Nordrehaug Åstrøm, Ingfrid Vaksdal Brattabø

**Affiliations:** 1 Oral Health Centre of Expertise in Vestland County, Bergen, Norway; 2 Institute of Clinical Dentistry, University of Bergen, Bergen, Norway

**Keywords:** Dentistry, Child abuse neglect, Oral health, Child welfare

## Abstract

**Background:**

The aptitude, knowledge, and competence of dental health personnel on child abuse and neglect (CAN) is not optimal for deciding when to file a report of concern to child welfare services (CWS).

**Objectives:**

The aim of this study was, firstly, to assess the association of the public dental health personnel ‘s (PDHP) training on CAN received in the last three work years, i.e., in 2016 through 2018 with filing reports to the CWS in the same period and secondly to assess the association of expressed need of training on CAN with filing reports to the CWS.

**Methods:**

This cross-sectional study uses data from an electronic survey census of PDHP from Norway (*n* = 1791) conducted in 2019. The Pearson chi-square test, non-parametric tests, logistic, and negative binomial regression were used for unadjusted and adjusted analysis. Data was reported with proportions, odds ratios (OR), incidence rate ratios and 95% confidence intervals (CIs).

**Results:**

From 2016 to 2018, the prevalence estimate of filing reports to CWS was 50%, with a mean (standard deviation) of 1.39 (2.11) reports sent. The logistic regression analysis showed an association between filing reports of concern and CAN training in the last three years. Compared to those that had not received CAN training during the three previous years, the ORs (95% CI) for filing reports to the CWS during the same period was 2.5 (1.6-4.0) for one day CAN work training, 3.2 (2.0-5.1) for 2–4 days CAN training and 4.9 (2.6–9.4) for five or more days CAN training. Compared to workers who did not need training in reporting (routines of CAN), those who expressed the need for a little more and more training were less likely to file a report. The corresponding OR were 0.6 (0.4–0.9) and 0.6 (0.3–0.9), respectively.

**Conclusion:**

CAN training during the last three years is associated with filing reports of concern to CWS in the same period among PDHP in Norway. The likelihood of filing CAN reports increased with the number of days of CAN training received. Secondly, the PDHP with an expressed need for training on CAN routines were less likely to report suspicions to CWS.

## Background

Child maltreatment or child abuse and neglect (CAN) encompasses any acts of commission or omission by a parent or other caregiver that result in harm, potential for harm, or threat of harm to a child (usually interpreted as up to 18 years of age), even if harm is not intended (the intended result) [1]. Globally, it was estimated that up to one billion children aged 2–17 years have experienced CAN as in physical, sexual, or emotional violence or neglect during the past year [2]. A systematic review of the prevalence of child sexual abuse in Nordic countries, published in 2016, found prevalence estimates of 3–23% among boys and 11–36% among girls [3]. According to the European status report on preventing child maltreatment, the prevalence of child sexual abuse was estimated at 13.4% and 5.7% in girls and boys, respectively [4]. The European report estimated the prevalence of physical and emotional neglect, reported as 16% and 18%, respectively. Applying these figures to the population of children in Europe suggests that 18 million children suffer from sexual abuse, 44 million from physical abuse and 55 million from mental abuse [4]. Recently, the national UVEVO [Ungdomsundersøkelsen om Erfaringer med Vold og Overgrep (Youth survey on exposure to violence and abuse)] study, assessing various forms of self-reported child maltreatment in Norwegian children up to adolescence, estimated the prevalence of sexual, physical, emotional and neglect to 28%,19%,18% and 14%, respectively [5]. In addition, the Norwegian Centre for Violence and Traumatic Stress conducted a national survey with over 4000 adult respondents, found that one in four adults reported being the victim of severe violence in their childhood home or sexual abuse in and/or outside the home during their childhood and thus may have carried the trauma burden into adulthood [6].The consequences of CAN include death, severe adversities or long-term impacts on childhood that might carry on into the later life course [7]. Exposure of children to violence and other hardships may result in negative coping and risky behaviours such as smoking, misuse of alcohol, abuse of drugs and engagement in risky sexual behaviour [8]. In addition, cognitive development can be negatively affected, resulting in educational and vocational underachievement [9]. Thus, it is necessary to prevent or avert probable CAN early on in childhood. The sixth strategy of the evidence-based technical package from WHO called “*INSPIRE: Seven strategies for ending violence against children”* deals with “Response services provision, for example, ensuring that children who are exposed to violence can access effective emergency care and receive appropriate psychosocial support” [10]. The public sector allows children to meet other adults like teachers, doctors, nurses and dentists besides those in their natural home environment. In addition, in Norway, it is mandatory for public dental health personnel (PDHP) to file suspected cases of CAN with child welfare services (CWS).

According to the Norwegian Public Health Act, minors up to 18 have a right to free dental healthcare services if they wish [11]. Therefore, the public dental health service can be a point of entry for protection authorities such as CWS to intervene as necessary if CAN is suspected. Moreover, as of 2021, Statistics Norway reports that 95.5% of children and youth (1–18 years) are under public supervision in the dental health service [12]. However, a recent international scoping review suggested that dental health workers’ aptitude, knowledge, and competence regarding issues of CAN are not optimal. Based on this scoping review of the literature, it has been recommended that dental personnel need continued CAN training to boost further competence in detecting and reporting [13].

The challenges of limited knowledge of CAN among PDHPs have been documented globally. In a study from Greece, a small proportion of dentists had ever received training on child protection at the undergraduate level and “having doubt about the diagnosis” was found to be the most common reason preventing a dentist from filing a case [14]. Whereas in Denmark, two studies done six years apart among dentists found similar results. The most frequently reported barriers to referral of CAN cases were uncertainty about observations, signs, and symptoms of CAN and referral procedures [15, 16]. Among Croatian dentists, a study found a lack of knowledge and uncertainty in recognising CAN cases [17]. In studies found among dental personnel from Oceania (Australia and New Zealand), a common barrier to reporting CAN was uncertainty in diagnosis [18, 19]. From the Middle East, similar findings of uncertainty in diagnosis and routines involved with reporting CAN have been reported among dental health workers at various levels [20–23]. The same lack of knowledge among dental personnel regarding issues of CAN has been documented in studies from Africa, India and the USA [24–28]. Even among the Norwegian PDHP with considerably higher rates of reporting CAN (60%) [29], one of the three causes identified for not reporting was insufficient knowledge of child maltreatment and reporting [30]. In addition, PDHP who had not received training on maltreatment and reporting to CWS during their professional education scored significantly higher on the barrier “insufficient knowledge of child maltreatment and reporting” than did dental personnel who had received such training [30].

Given the detrimental effects of CAN on childhood and the potential spillover effects across the life course and to later generations, it is important to explore the relationship of CAN training with dental workers’ ability to file reports of concern. In addition, the dimension of need of training and the quantity of it is lacking and is necessary to try to understand further and could further inform the planning of training activities and possibly explain the reporting behaviour of the PDHP. Most of the literature has assessed dental workers’ knowledge and attitudes on CAN. However, there are few studies in the literature that have explored the association of CAN education received or expressed the need for education about CAN topics among dental health workers to improve their reporting capabilities.

The purpose of this study was firstly to assess the association of the (PDHP) received training in CAN during the last three years (2016 through 2018) with filing reports to CWS during the same period. Secondly, this study assesses the association of the expressed need for training on CAN with filing CAN reports to the CWS. The hypothesis was that received training and expressed need for training in CAN were associated with more- and less filing of reports, respectively.

## Methods

This cross-sectional study uses data from an electronic survey targeting PDHP of Norway in 2019 as part of the ‘Children at risk and oral health research project’; childrenatriskandoralhealth.com. A census of public dental hygienists and dentists (*n* = 1791) from all counties in Norway were invited to participate and received the survey. The chief of the public dental health service provided the names and email addresses of the PDHPs and permitted them to participate during working hours. The PDHPs received informed consent and a cover letter about the study by a link sent to their respective email addresses. A total of 1270 dental health care workers accepted to participate (response rate 1270/1791, 70.9%). The participants who responded and answered more than ten questions were 69% (1238/1791) and constituted the analytical sample.

Ethics approval for the study was given by ethics committees of the region called regional ethical committee (reference number: 2018/2523/REK nord), Norwegian Social Science Data Services (NSD) and the ombudsman of Hordaland County (reference number 364,916 NSD). Informed consent to participate was taken from all participants. The NSD was responsible for the distribution and collection of the electronic questionnaire.

The questionnaire was piloted in one county, among 176 dentists and dental hygienistsand then utilised in a national survey among dental health personnel in Norway in 2014 [31]. The pilot study was performed to test the questionnaire and its content, the technical issues regarding the questionnaire’s distribution and functioning and the survey’s implementation process. From the results of the pilot study, extra efforts were made to minimise error terms of non-response and measurement error. Details of the pilot study have been previously published [31].

### Outcome variable

The outcome variable was filing a report of concern about suspicion of CAN in the last three years (2016 through 2018). This variable was assessed by first asking: “Have you ever filed a report of concern in your career in the public health dental health service?”. The responses were yes (1) or no (0). A follow-up question for those who responded “yes” was, “Have any of these reports of concern been filed in 2016 through 2018?”. The responses were yes (1) or no (0). An additional follow-up question (for those who confirmed having filed a report of concern during 2016 through 2018 was, “How many reports of concern have you sent from the period of 2016 through 2018?” The responses ranged from one up to ten and more. If more than 10, they were asked to indicate the number.

Due to the layout of the questionnaire, a new variable, including reporting behaviour for all respondents in the period 2016–2018, was created. Respondents who had not sent a report of concern during their career and respondents who had not sent a report of concern 2016–2018 were registered with 0 reports of concern in the last 3 years 2016–2018. These were merged with the respondents having reporting experience 2016–2018, being registered with the number of reports sent for the three-year period.

### Main exposure variables

Another question assessing seminars about CAN during work (at graduate or post-graduate level) in the public dental health service was, From 2016 to 2018, “have you in connection to your work in the public dental health service been part of any training/courses/seminars in relation to CAN?”. The response categories were yes, 1 day or less (1), yes, 2–4 days (2), yes, 5 days or more (3), no (4)and I do not know (5).

Two questions assessed the expressed need for more training regarding reporting routines of CAN as well as the need for more training in CAN topics, i.e., “Do you have a need for more training on routines for filing reports of CAN to CWS?” The responses were no need for more training (1), not sure (2), yes, have need for little more training (3), yes, have need for more training (4), yes, have need for much more training (5). The need for more training in the topic of child abuse and neglect was assessed by the question; “Do you have a need for more training on the topic of CAN?” Response categories were no need for more training (1), not sure (2), yes, have need for little more training (3), yes, have need for more training (4), yes, have need for much more training (5).

### Independent covariates (potential confounding variables and other covariates)

The choice of variables was made based on socio-demographic characteristics (sex, age and position held in the PDHS) and variables from previous works (working experience, number of patients attending the public clinics in a year, number of those employed in the clinic, geographical location and number of residents in the municipality) on the CAN found to be independently associated with filing reports of concern regarding CAN [29]. The overview of these variables is summarised in Table 1.

**Table 1:** Frequency distribution of characteristics of participants (*n* = 1238)

Variables	Number analysed.	Frequency distribution
Independent variables and potential confounding	*n*	*n* (%)
**Gender**	1102	
Female		945 (85.8)
Male		157 (14.2)
**Age**	1101	
20-39yrs		579 (52.6)
40+		522 (47.4)
**Position held**	1238	
Dental hygienist		397 (32.1)
Dentist		824 (66.6)
Other (psychologists, dental secretary)		17 (1.4)
**Number of employees in the Clinic**	1102	
1–10		492 (44.6)
11–20		610 (55.4)
**Work experience in dental public service**	1207	
1-10years		641 (53.1)
11+		566 (46.9)
**Number patients seen in last year**	1084	
0–500		384 (35.4)
>500		700 (64.6)
**Number of residents in the municipality**	1099	
0–10,000		329 (29.9)
10,001–40,000		397 (36.1)
40,001+		373 (33.9)
**Geographical Region**	1043	
North (Finnmark, Troms and Nordland)		162 (15.5)
Central (Trøndelag and Møre og Romsdal)		154 (14.8)
West (Sogn og Fjordane, Hordaland and Rogaland)		265 (25.4)
South (Vest Agder, Aust Agder, Telemark, Vestfold, and Buskerud)		198 (19.0)
East (Akershus, Oppland, Hedmark, Østfold and Oslo)		264 (25.3)
**CAN education**		
Received education on CAN during undergraduate education	1178	
I do not know		121 (10.3)
No		333 (28.3)
Yes, for 1 day or less		241 (20.5)
Yes, for 2–4 days		344 (29.2)
Yes, for 5 days or more		139 (11.8)
**Main exposure variables**		
Received CAN training seminars in 2016 through 2018	1178	
I do not know		37 (3.1)
No		164 (13.9)
Yes, for 1 day or less		408 (34.8)
Yes, for 2–4 days		483 (41.0)
Yes, for 5 days or more		86 (7.3)
Need for more training seminars on topic of CAN	1177	
No not needed		102 (8.7)
Not sure		115 (9.8)
Yes, I need little more training		406 (34.5)
Yes, I need more training		443 (37.6)
Yes, I need a lot more training		111 (9.4)
Need more training on routines of reporting CAN	1177	
No not needed		214 (18.2)
Not sure		147 (12.5)
Yes, I need little more training		421 (35.8)
Yes, I need more training		328 (27.9)
Yes, I need a lot more training		67 (5.7)

*For number that do not add up to 1238 indicate missing data

Measures assessing socio-demographic characteristics assessed were age, genderand occupation. Specifically, the age was assessed in six age group categories (20–29,30–39,40–49,50–59,60–69,70 + years). In the analysis, the age groups were recategorised to two groups:20–39 (1) and 40+ (2). The gender was assessed as either a woman (1) or a man (2). The occupation was assessed by, “What is your current position in the dental health service?” The respective responses were dental hygienist (1), dental hygienist with a leadership role (2), dentist (3), dentist with a specialisation (4), dentist with a leadership role (5), psychologist (6)and others (7). For the analysis, the groups involving dentists and hygienists were selected: dentists (1) and dental hygienists (2). the other positions were not included in the analysis.

Based on previous work [29], variables independently associated with CAN, such as personal characteristics (work experience), organisational characteristics (number of patients treated in the last 12 months)and external characteristics (number of residents in the municipality, geographical region/county of work) were assessed. The work experience was assessed by asking, “How many years have you been employed in the public dental health service?” The responses were categorised as from 1 to 10 years (1) and 11 years + (2). The number of patients treated in the last 12 months by asking, “approximately how many patients under 18 years have you examined or treated in the last 12 months?” The response options were 1-250 (1), 251–500 (2) 501–750 (3),751–1000 (4), 1001–1250 (5), 1251–1500 (6), 1501+ (7) and 0 (8). For analysis, these response categories were dichotomised to 0-500 (0) and 500+(1). The size of the municipality was assessed by the question how many residents there are in the municipality where the dental clinic is located. The response categories ranged from 0 to 500 (1), 501-10000 (2), 10,001–15,000 (3), 15,001–20,000 (4), 20,001–40,000 (5), 40,001–80,000 (6) 80,001+ (7). These were recategorized into three categories 0-1000(0). 10,001–40,000(1) and 40,001+(2). The geographical location where the work is located was assessed by asking which county you are employed. The response options were 19: Oppland (1), Hordaland (2), Rogaland (3), Nordland (4), Troms (5), Finnmark (6), Møre og Romsdal (7), Sogn og Fjordane (8), Vest Agder (9), Aust Agder (10), Telemark (11), Buskerud (12), Hedmark (13), Vestfold (14), Østfold (15), Oslo (16), Akershus (18) and Trøndelag (19). In the analysis the counties were recategorized into five geographical regions namely, south (VestAgder, AustAgder, Telemark, Vestfold and Buskerud), central (Trøndelag and Møre og Romsdal), west (Sogn og Fjordane, Hordaland and Rogaland), north (Finnmark, Troms and Nordland), east (Oppland, Hedmark, Østfold, Akershus and Oslo.).

Education /training about CAN was assessed by asking about training concerning CAN during school at undergraduate level and extra training seminars during work in the public dental health service at postgraduate level using the following questions: “During your education (at undergraduate), did you receive any training about CAN?” The response categories were yes, 1 day or less (1), yes, 2–4 days (2), yes, 5 days or more (3), no (4)and I do not know (5).

The data was analysed using IBM Statistical Package for Social Sciences version 28 (SPSS Inc., Chicago, IL). The sample characteristics were described using means and standard deviations for continuous variables, while categorical ones were summarised as proportions. Crude analysis was performed using the chi-square test for categorical variables and non-parametric tests, Mann-Whitney and Kruskal-Wallis for continuous variables at 0.05 significance level. Regression analysis using logistic regression for binary outcomes and negative binomial analysis for over-dispersed count variables. The estimates were reported using odds ratios (OR), incidence rate ratios (IRRs) and confidence interval (CI) at 95%. The main exposure variables, training seminars on CAN in the last three years (2016 through 2018) and the need for training on CAN, were regressed on the outcome: filing reports of concern in the previous three years (2016 through 2018). The possible confounding variables were socio-demographics and relevant exposure variables, as documented in earlier works and described aboveand having received CAN education at the undergraduate level.

## Results

### Descriptive characteristics

Table 1 summarises the sample characteristics among all the respondents in the survey. The majority of the participants were female (86%). The most common position held was that of a dentist (67%), followed by dental hygienists (32%), then psychologist and dental secretary (1.4%). More than half the respondents (53%) had at least ten years of working experience. Geographically, most of the respondents were from the western (25%) and the eastern (25%) regions, followed by the south (19%), north (16%)and lastly, the central (15%) region. Sixty-two per cent of the PDHPs had received education on CAN during their education at the undergraduate level. While 83% had received seminars during work on CAN in the three years 2016–2018. The need for more training seminars on CAN and the routines of filing CAN reports was expressed by 82% and 69% of the respondents, respectively.

### The proportion of filed reports and mean number of suspected CAN reports sent to the CWS by PDHS

Table 2 shows that 70% had ever filed a report of concern to the CWS in their work career in the public dental health service in line with previous published work. The corresponding figure for having filed a report from 2016 to 2018 was 50%. The mean number (standard deviation) of reports of CAN sent to CWS during the career by the PDHPs was 3.04 (1.39), while corresponding figures for reports sent from 2016 to 2018 were 1.39 (2.11).

**Table 2:** Proportion of public dental health personnel that have filed a report of concern of child abuse and neglect (CAN) to child welfare services and mean number of CAN reports during their entire career, and between 2016–2018

	During career in PDHS	Between 2016–2018 in PDHS
	***n*** (%)	***n*** (%)
Proportion of PDHP that have filed reports to CWS		
Not filed report	366 (29.6)	611 (49.6)
Yes, filed report	865 (69.9)	620 (50.4)
	**Mean (standard deviation)**	**Mean (standard deviation)**
Number of reports filed for suspicion of CAN to CWS	3.04 (1.39)	1.39 (2.11)

### Association of proportion of filed reports of concern to CWS in 2016 through 2018 with socio-demographic characteristics, CAN training in 2016 through 2018 and expressed need for training

Table 3 depicts the crude and adjusted logistic associations showed by the frequency, percentage distribution and odds ratios (95% Cls) of filing reports of concern to CWS in 2016 through 2018 by socio-demographic variables, CAN training seminars in 2016 through 2018 and expressed the need of CAN training. The results showed significant unadjusted associations of filing reports of concern in 2016 through 2018 with gender, position held, working experience, number of patients seen in the last year, number of residents in the municipality, geographical region and receipt of CAN training in 2016 through 2018. Adjusted analysis using logistic regression of filing a report of CAN in 2016 through 2018 by the variables found to be significant in unadjusted analysis revealed that gender, the number of patients seen, geographical region, CAN training seminars in 2016 through 2018 and need for more education on routines to be independently associated. In addition, for those who were aware of how many days they received CAN training seminars, the more days of CAN training seminars received, the more likely the filing of CAN reports to CWS. Specifically, the ORs (CI 95%) estimates for those who had received training seminars about CAN in 2016 through 2018 for one day, 2–4 days and five days or more were 2.5 (1.6-4.0)3.2 (2.0-5.1) and 4.9 (2.6–9.4), respectively. In the categories of the “need for more training on routines of CAN”, those who reported the need for a little more training and more training were less likely to have filed a report of concern in 2016 through 2018 with ORs (CI 95%) estimates of 0.6 (0.4–0.9) and 0.6 (0.3–0.9), respectively.

**Table 3:** Unadjusted analysis and adjusted logistic regression of filing a report of concern of child abuse and neglect (CAN) or not between 2016 through 2018 (*n* = 1231)

Variables	Did not file a report of concern	Filed a report of concern	Logistic regression analysis
	***n*** (%)	***n*** (%)	
**Gender**			
Female	469 (49.9)	470 (50.1) **	Ref
Male	99 (63.5)	57 (36.5)	0.5 (0.4–0.8) *
**Age**			
20-39yrs	284 (49.4)	291 (50.6)	
40+	284 (54.7)	235 (45.3)	
**Position held**			
Dental hygienist	178 (44.9)	218 (55.1) *	Ref
Dentist	421 (51.3)	399 (48.7)	0.9 (0.7–1.3)
**Number of employees in the Clinic**			
1–10	269 (54.8)	222 (45.2)	
11–20	299 (49.5)	305 (50.5)	
**Work experience in dental public service**			
1–10 years	337 (53.2)	297 (46.8) *	Ref
11 + years	262 (46.3)	304 (49.2)	1.0 (0.7–1.3)
**Number patients seen in last year**			
0 -500	228 (59.4)	156 (40.6) **	Ref
>500	331 (47.3)	369 (52.7)	1.5 (1.1–2.1) *
**Number of residents in the municipality**			
0–10,000	192 (58.5)	136 (41.5) *	Ref
10,001–40,000	186 (47.3)	207 (52.7)	1.4 (1.0–2.0)
40,001	188 (50.7)	183 (49.3)	1.2 (0.8–1.6)
**Geographical Region**			
North (Finnmark, Troms and Nordland)	74 (45.7)	88 (54.3) **	Ref
Central (Trøndelag and Møre og Romsdal)	99 (64.7)	54 (35.3)	0.4 (0.3–0.7) **
West (Sogn og Fjordane, Hordaland and Rogaland)	154 (58.1)	111 (41.9)	0.5 (0.3–0.7) **
South (Vest Agder, Aust Agder, Telemark, Vestfold, and Buskerud)	94 (47.5)	104 (52.5)	0.7 (0.4–1.1)
East (Akershus, Oppland, Hedmark, Østfold and Oslo)	122 (46.7)	139 (53.3)	0.7 (0.5–1.1)
**CAN education**			
Received education on CAN during undergraduate education			
No	176 (53.0)	156 (47.0)	Ref
Do not know	72 (59.5)	49 (40.5)	0.7 (0.4–1.2)
Yes, for 1 day or less	126 (52.7)	113(47.3)	1.2 (0.8–1.7)
Yes for 2–4 days	164 (48.1)	177 (51.9)	1.4 (1.0-2.1)
Yes for 5 days or more	68 (49.3)	70 (50.7)	1.0 (0.6–1.7)
Received CAN training seminars in 2016 through 2018			
No	120 (74.5)	41 (25.5) **	Ref
Do not know	21 (56.8)	16(43.2)	2.8 (1.2–6.6) *
Yes, for 1 day or less	213 (52.6)	192 (47.4)	2.5 (1.6-4.0) **
Yes for 2–4 days	220 (45.6)	262 (54.4)	3.2 (2.0-5.1) **
Yes for 5 days or more	32 (37.2)	54 (62.8)	4.9 (2.6–9.4) **
Need more training seminars on the topic of CAN			
No, not needed	53 (53.0)	47 (47.0)	Ref
Not sure	68 (59.1)	47 (40.9)	0.9 (0.4–1.7)
Yes, I need little more training	199 (49.5)	203 (50.5)	1.6 (0.9–2.9)
Yes, I need more training	229 (51.7)	214 (48.3)	1.7 (0.9–3.1)
Yes, I need a lot more training	57 (51.8)	53 (49.2)	1.9 (0.8–4.2)
Need more training on routines of reporting CAN			
No, not needed	97 (45.8)	115 (54.2)	Ref
Not sure	69 (46.9)	78 (53.1)	0.9 (0.6–1.6)
Yes, I need little more training	223 (53.3)	195 (46.7)	0.6 (0.4–0.9) *
Yes, I need more training	178 (54.4)	149 (45.6)	0.6 (0.3–0.9) *
Yes, I need a lot more training	39 (59.1)	27 (40.9)	0.4 (0.2-1.0)

### Association of mean number of reports of concern sent to CWS with socio-demographic characteristics, CAN training in 2016 through 2018 and expressed need for training

Table 4 shows the crude associations and adjusted negative binomial regression of mean number of reports of CAN filed to CWS in 2016 through 2018 with social demographic variables, CAN training seminars in 2016 through 2018 and expressed need for more CAN training. Gender, position held, number of employees, work experience, number of patients seen in a year, number of residents in municipality, geographical regionand receipt of CAN training seminars in 2016 through 2018 showed statistical significance in the unadjusted analysis. In the negative binomial regression, those that received CAN training seminars in 2016 through 2018 had a statistically significant relative mean number of reports with IRR and (95% CIs) of 2.42 (1.72–3.41) for training CAN seminars for one day, 2.59 (1.84–3.65) for training CAN seminars for 2–4 days and 3.56 (2.31–5.51) for training CAN seminars of 5 days or more. The other significant covariates were the number of patients seen in a year, number of residents in the municipality geographical regionand the expressed need for more training on CAN and routines of filing CAN reports.

**Table 4:** Unadjusted analysis and adjusted negative binomial regression of mean number of reports of a CAN filed between 2016 through 2018 (*n* = 1231)

Variables	Mean reports filed (SD)	Neg binomial analysis
**Gender**		
Female	1.29 (1.90) *	Ref
Male	0.94 (1.78)	0.78 (0.59–1.03)
**Age**		
20-39yrs	1.32 (1.20)	
40+	1.15 (1.75)	
**Position held**		
Dental hygienist	1.59 (2.10) *	Ref
Dentist	1.31 (2.12)	0.94 (0.77–1.14)
**Number of employees in the Clinic**		
1–10	1.13 (1.95) *	Ref
11–20	1.34 (1.83)	0.96 (0.78–1.19)
**Work experience in dental public service**		
1–10 years	1.18 (1.82) *	Ref
11 + years	1.59 (2.36)	1.06 (0.85–1.31)
**Number patients seen in last year**		
0–500	0.86 (1.37) **	Ref
>500	1.47 (2.09)	1.53 (1.24–1.90) **
**Number of residents in the municipality**		
0–10,000	0.94 (1.73) *	Ref
10,001–40,000	1.33 (1.87)	1.33 (1.01–1.77) *
400,001	1.42 (2.01)	1.34 (0.93–1.64)
**Geographical Region**		
North (Finnmark, Troms and Nordland)	1.32 (1.74) **	Ref
Central (Trøndelag and Møre og Romsdal)	0.75 (1.27)	0.56 (0.40–0.79) *
West (Sogn og Fjordane, Hordaland and Rogaland)	0.94 (1.49)	0.58 (0.43–0.79) *
South (Vest Agder, Aust Agder, Telemark, Vestfold, and Buskerud)	1.41 (1.87)	0.91 (0.67–1.23)
East (Akershus, Oppland, Hedmark, Østfold and Oslo)	1.64 (2.49)	0.96 (0.72–1.30)
**CAN education**		
Received education on CAN during undergraduate education		
No	1.13 (1.59)	Ref
I do not know	1.09 (1.81)	0.86 (0.61–1.21)
Yes, for 1 day or less	128 (2.26)	1.15 (0.88–1.50)
Yes for 2–4 days	1.26 (1.67)	1.17 (0.90–1.52)
Yes for 5 days or more	1.59 (2.26)	1.30 (0.94–1.79)
Received CAN training seminars in 2016 through 2018		
No	0.46 (0.90) **	Ref
I do not know	1.14 (2.32)	2.79 (1.55–5.02) **
Yes, for 1 day or less	1.22 (1.85)	2.42 (1.72–3.41) **
Yes for 2–4 days	1.42 (1.96)	2.59 (1.84–3.65) **
Yes for 5 days or more	1.94 (2.23)	3.56 (2.31–5.51) **
Need more training seminars on the topic of CAN		
No not needed	1.17 (1.92)	Ref
Not sure	1.01 (1.48)	0.10 (0.70–1.73)
Yes, I need little more training	1.17 (1.80)	1.46 (0.99–2.17)
Yes, I need more training	1.35 (1.97)	1.72 (1.45–2.59) *
Yes, I need a lot more training	1.45 (2.08)	2.11 (1.25–3.56) *
Need more training on routines of reporting CAN		
No not needed	1.63 (2.40)	Ref
Not sure	1.19 (1.55)	0.70 (0.50–0.97)
Yes, I need little more training	1.11 (1.68)	0.58 (0.45–0.78) **
Yes, I need more training	1.22 (1.79)	0.62 (0.45–0.84) *
Yes, I need a lot more training	1.18 (2.13)	0.48(0.28–0.83) *

## Discussion

This study explored the association of receipt of CAN training in the last three work years 2016 through 2018 and expressed the need for CAN with filing reports of concern to the CWS from 2016 to 2018.

This national survey, including a census of PDHP from Norway, found that it was more likely to file reports of concern to CWS during 2016–2018 for those who had training seminars on CAN in the same period. The likelihood of filing reports of concern was high with 1 day, higher with 2–4 days and highest with 5 days or more with more days of CAN training seminars received. In addition, those who expressed a little more or a lot more need of training on routines for reporting CAN to CWS were less likely to file reports of concern to the CWS. Conversely, those who expressed little need, more need and a lot more need for training on CAN topics reported more though not significantly than those who expressed that they did not need CAN training.

The nationally representative nature and the high response rate are obvious strengths of this study. As the PDHP from all counties of Norway had the opportunity to participate, the present findings may be regarded as representative to the PDHP of Norway. There are some limitations that must be acknowledged. Notably, a cross-sectional study design methodology, does not consider the temporal dimensions of causality. It cannot be ascertained whether having had the CAN training seminars in the last there years was a cause or a consequence of filing of reports of concern to child welfare services. Thus, we can only document the association of receiving CAN training in the last three years (2016 through 2018) with filing reports of concern to CWS and not the effect of the CAN training in the previous three last year’s as being causal to filing reports of concern to child welfare services. There is also the issue of recall bias that relies on the respondents memory to recollect information or some PDHP that have reported before could have recollected less than their rookie colleagues and this could lead to biased unequal groups [32]. However, filing these kinds of reports to CWS is a serious decision so it can be assumed that the respondents will not easily forget the events or at least with feedback from CWS on the cases reported the PDHP might be able to recall optimally. Also, social desirability bias could have been responsible for some of the CAN trained PDHP reporting that they filed reports to indicate that they are performing as expected Thus, may have lead to overestimated odds ratios.

Similar to our findings, a study from the UK found higher rates of post-graduation child protection training in later years were associated with substantial improvement in reduction of suspecting and failing to file a report to authorities [33]. However, the former study contrary to our study targeted specialised paediatric dentists, included attitudinal factorsand had a 64% response rate while our study included all PDHP of Norway although did not include attitudinal measures in the analysis. It has been shown that the dental care providers with specialist roles such as paediatric dentists tend to report more since they are more confident and knowledgeable [34]. In contrast, it has been shown that although paediatric dentists tend to have good expertise as relates with oral heath there are disparities in CAN knowledge when compared to other professionals like doctors and nurses [35]. Another finding from our study indicated that those who expressed little or much need for CAN training on routines for filing reports were less likely to file reports of concern to CWS A logical explanation for this trend as summarised from a recent literature review is that lack of knowledge or uncertainty of referral procedures after a diagnosis of CAN is associated with less reporting [13].

Arguably, child protection or CAN training is a key factor for aiding dental health workers in diagnosing of CAN and subsequent filing of reports, however, our findings did not show an association of filing reports of concern with undergraduate CAN training, as could have been expected. It can be speculated that CAN training has become more regular in the education institutions in the recent past than before and it may not have been sufficient for the older generations of dental personnel. According to Bradbury et al., adjunctive CAN training improves reporting in addition to undergraduate CAN continued training [13]. Albeit, it was more likely to file reports of concern with CAN training in the previous three years. These findings indicate that it is of importance to focus on routine training about CAN regularly. Our findings show that continuing education on CAN in recent years increases dental health personnel’s likelihood of exercising their duty to file suspected cases of CAN to the CWS. These findings corroborate with results from a similar web based survey from Finland among dental professionals which found that dental care workers with postgraduate training and graduate training in CAN were often times most likely to report i.e. 2.4 times 1.9 times respectively than those without any training [36].This result may further emphasize the importance of continued training at the graduate level or continued dental education on top of the training received at the undergraduate level. Interestingly, from a smaller survey among Italian dentists with CAN education that found positive results with training at undergraduate level. The former study involved simulation of clinical cases using photos and found a positive association as the educated dentists in the study correctly answered when cases were depicted that were compatible with CAN [37]. However, this result was not in regular clinical practice scenario and the participants in the group that had received CAN education were skewed than the group that had not received CAN training.

The duty of dental health providers regarding mandatory filing of suspicions of CAN is clear as required by the law and is echoed in the literature [11, 38]. The complexities therein of deciding to report or not to report cannot be reduced to having CAN training only [30, 39]. Other important intricate interconnecting factors like socio-demographical characteristics, individual attitudes and external factors come into play and have been discussed in previous work [29, 40]. Our findings corroborated with previous published articles showing that gender, working experience, number of patients seen in the last year, the number of residents in the municipality and geographical region were also important determinants of filing reports of concern. Thus, we refer to those studies as cited above.

The findings from the negative binomial regression were similar those of the logistic regression analysis discussed above in terms of interpretation. Briefly the relative incidence rate ratios for filing reports of concern were more with more days of receipt of CAN training in the previous three last years. Conversely, those that expressed a need for CAN training on routines were less likely to file reports of concern. These findings may indicate that the confidence to file reports of concern improves with training and more so with increased days of CAN training received. On the contrary the finding that PDHP who expressed need for more CAN training were more likely to report might indicate that detection of CAN is severe, complexand challenging, as each child and contest is uniqueand that PDHP having reported to CWS experiences these challenges and acknowledges that they need more knowledge. It can only be speculated that the more PDHP work with CAN, the more they understand as well get more confident with reporting routines as they are set and easier to follow and learn. In addition, setting aside sufficient time for the PDHP to have CAN training seminars apart from the work schedule may be beneficial to increase the confidence to file reports or improve the knowledge of filing routines to CWS.

Further interventional research is needed, particularly appreciating the dimensionality and complexities that the public dental health providers navigate when faced with CAN suspicions. Furthermore, more studies need to investigate the interdisciplinary relationships between dental health providers and the relevant authorities like CWS to capture the gaps that exist with regards to procedures or routines of reporting documented in the literature.

## Conclusion

Child abuse and neglect training during last three years is associated with having filed reports of concern to CWS in the last three years among PDHP of Norway. The likelihood of filing reports of CAN increased with the number of days of CAN training received. Secondly, the PDHP with expressed need for training on CAN routines were less likely to report suspicions to CWS.

## Data Availability

The datasets generated and/or analyzed during the current study are not publicly available due on-going analyses articles are to be published from the same dataset but are available from the corresponding author on reasonable request.

## Abbreviations

CAN: Child Abuse and Neglect
CWS: Child Welfare Services
NSD: Norwegian Social Science Data Services
PDHP: Public Dental Health Personnel
UVEVO: Ungdomsundersøkelsen om Erfaringer med Vold og Overgrep (Youth survey on exposure to violence and abuse)
